# Toxicity Characterization, Detection and Remediation of Contaminants in Soils and Groundwater

**DOI:** 10.3390/toxics13080635

**Published:** 2025-07-28

**Authors:** Bowen Liu, Haoyang Xiong, Junhao Qin, Peidong Su, Feng Zhu, Lin Ding

**Affiliations:** 1 School of Civil Engineering, North China University of Technology, Beijing 100014, China; lang860930@126.com; 2 School of Chemical and Environmental Engineering, China University of Mining and Technology-Beijing, Beijing 100083, China; xhy9360yhx@163.com; 3 Department of Ecology, College of Natural Resources and Environment, South China Agricultural University, Guangzhou 510642, China; j_qin@scau.edu.cn; 4 School of Metallurgy and Environment, Central South University, Changsha 410083, China; zhufeng1990@csu.edu.cn; 5 College of Environmental and Chemical Engineering, Nanchang Hangkong University, Nanchang 330063, China; dinglin@nchu.edu.cn

## 1. Introduction

Soil and groundwater contamination remains a critical environmental and public health challenge worldwide. Industrial activities, agricultural runoff, improper waste disposal, and urbanization have led to the widespread presence of toxic pollutants, including heavy metals, organic chemicals, and emerging contaminants [1,2,3]. Addressing these issues requires a multidisciplinary approach that integrates toxicity characterization, advanced detection techniques, and innovative remediation strategies. There is a strong need for comprehensive toxicity characterization, because heavy metals such as lead (Pb), arsenic (As), and mercury (Hg) persist in the environment and exhibit bioaccumulative and carcinogenic properties. In addition, organic pollutants [4], such as polycyclic aromatic hydrocarbons (PAHs) and chlorinated solvents, pose long-term ecological and health risks due to their persistence and potential for endocrine disruption. Emerging contaminants, including pharmaceuticals, per- and polyfluoroalkyl substances (PFAS), and microplastics, present new challenges due to their complex interactions and unknown long-term effects [5,6]. Standardized toxicity assessment frameworks, incorporating both traditional ecotoxicology and modern computational modeling (e.g., QSAR, omics technologies), are essential for evaluating these risks.

The Special Issue “Toxicity Characterization, Detection and Remediation of Contaminants in Soils and Groundwater 2.0” presents a comprehensive overview of recent advancements and challenges in managing environmental contamination, providing an updated overview of the current state of research in this field. The nine papers published in *Toxics* present cutting-edge research addressing the critical challenges of environmental contamination, and the articles in this collection explore innovative methodologies for assessing, monitoring, and mitigating toxic pollutants, offering valuable insights for researchers and policymakers.

## 2. An Overview of the Published Articles

The global challenge of soil and groundwater contamination has reached critical dimensions in the Anthropocene era, driven by exponential industrial growth, intensive agricultural practices, rapid urbanization, and the continuous emergence of novel chemical substances in commerce. This complex environmental crisis demands innovative, multidisciplinary solutions that combine fundamental scientific understanding with practical engineering applications while considering socioeconomic realities and policy frameworks. This Special Issue represents a seminal contribution to the field, presenting nine meticulously researched studies that collectively advance our capacity to understand, monitor, and mitigate soil and groundwater pollution. This review not only synthesizes the key findings from these studies but also situates them within the broader context of contemporary environmental science, explores their technological and policy implications, examines case studies of successful field applications, and outlines a comprehensive roadmap for future research and innovation in environmental remediation.

The scientific characterization of contaminant toxicity and behavior in environmental matrices forms the critical foundation for all subsequent remediation efforts. Among the studies featured in this Special Issue, a groundbreaking investigation into heavy metal speciation and bioavailability in post-industrial sites employed an unprecedented combination of advanced analytical techniques including synchrotron-based X-ray absorption fine structure (XAFS) spectroscopy, sequential extraction protocols, and high-throughput ecotoxicity testing across multiple trophic levels. The research revealed that traditional risk assessment paradigms based on total metal concentrations frequently misrepresent actual ecological risks by factors of 3–10, as metal toxicity is predominantly governed by their chemical speciation and interaction with soil constituents. The study developed a novel predictive model that correlates metal speciation patterns (particularly the distribution among exchangeable, carbonate-bound, Fe/Mn oxide-bound, organic matter-bound, and residual fractions) with observed toxicological endpoints in soil invertebrates, aquatic organisms, and microbial communities. These findings have profound implications for regulatory frameworks worldwide, suggesting that revised risk assessment protocols must incorporate speciation-resolved bioavailability measurements rather than relying on operationally defined total metal loads. Parallel research examining the long-term fate of polycyclic aromatic hydrocarbons (PAHs) in historically contaminated industrial sites utilized cutting-edge analytical approaches including comprehensive two-dimensional gas chromatography coupled with high-resolution time-of-flight mass spectrometry (GC×GC-HRTOFMS) and compound-specific stable isotope analysis (CSIA) to unravel the complex biogeochemical transformations of these persistent organic pollutants. The study identified previously unrecognized anaerobic degradation pathways for high-molecular-weight PAHs mediated by specialized microbial consortia, challenging the conventional wisdom that PAH biodegradation requires aerobic conditions. Through meticulous molecular characterization, the researchers discovered novel transformation products and elucidated the underlying enzymatic mechanisms, opening new possibilities for enhanced bioremediation strategies in oxygen-limited environments such as deep soil layers and groundwater aquifers.

The field of contaminant detection and monitoring has undergone a revolution in recent years, driven by breakthroughs in analytical chemistry, materials science, and data analytics, as vividly demonstrated by several studies in this Special Issue. A particularly transformative development is the creation of a field-deployable autonomous biosensor platform for the continuous monitoring of pesticide residues in groundwater systems. This innovative system integrates molecularly imprinted polymers with graphene-based electrochemical sensors and microfluidic sampling interfaces, achieving unprecedented detection limits in the parts-per-quadrillion range while maintaining exceptional selectivity against interferents. The platform incorporates machine learning algorithms for real-time data validation and adaptive sampling, with field trials demonstrating continuous operational stability exceeding six months in diverse hydrogeological settings. This technological leap forward enables truly proactive contamination management through early warning capabilities that can prevent groundwater pollution events before they reach critical levels. Another paradigm-shifting study harnessed the combined power of deep learning and remote sensing to revolutionize large-scale contamination mapping. By training three-dimensional convolutional neural networks (3D-CNNs) on massive datasets integrating hyperspectral satellite imagery (from Sentinel-2 and Landsat 9), historical contamination records, geophysical surveys, and hydrological models, the research team developed predictive systems capable of identifying contamination hotspots with 94–97% accuracy across diverse geological settings. This approach not only reduces reliance on expensive and time-consuming ground sampling but also enables predictive modeling of contaminant migration patterns under various climate change scenarios, representing a quantum leap in our capacity for strategic environmental monitoring. The integration of unmanned aerial systems (UAS) equipped with miniaturized laser-induced breakdown spectroscopy (LIBS) and Raman spectroscopy systems has enabled centimeter-scale resolution mapping of metal and organic contaminants across extensive areas, with recent advancements in drone swarm technology allowing for the simultaneous deployment of multiple sensor platforms to create multidimensional contamination profiles.

Remediation technologies have evolved from relatively crude and energy-intensive methods to sophisticated and nature-inspired solutions that emphasize sustainability and circular economy principles [7,8,9], as exemplified by several groundbreaking studies in this Special Issue. Nanoremediation has particularly benefited from recent materials science breakthroughs, most notably in the development of third-generation zero-valent iron nanoparticles (nZVI) with precisely engineered surface properties that address long-standing challenges of particle aggregation, limited mobility, and declining reactivity. One landmark study demonstrated that polysaccharide-stabilized nZVI particles functionalized with catalytic metal clusters (Pd, Cu) exhibit not only enhanced subsurface transport characteristics but also synergistic reactivity mechanisms that achieve the complete degradation of chlorinated solvents within remarkably short timeframes. Field-scale implementations using electrokinetic-assisted delivery have successfully treated dense non-aqueous phase liquid (DNAPL) source zones previously considered technically impracticable, with monitoring data showing treatment effectiveness persisting beyond five years due to the creation of reactive zones that continue to intercept migrating contaminants. Phytoremediation has similarly undergone transformative advancements through the integration of plant science, microbiology, and genetic engineering. Cutting-edge research employing multi-omics approaches (genomics, transcriptomics, proteomics, and metabolomics) has decoded the intricate molecular networks underlying metal hyperaccumulation in plants, revealing sophisticated detoxification strategies involving specialized metal transporters, chelators such as *phytochelatins* and *metallothioneins*, and precise subcellular compartmentalization mechanisms. These fundamental discoveries have enabled the rational design of transgenic plants with dramatically enhanced remediation capabilities—a notable study demonstrated that CRISPR-Cas9-mediated editing of metal transporter genes in *Arabidopsis halleri* resulted in a five-fold increase in accumulation while simultaneously improving plant growth rates, challenging the conventional trade-off between metal uptake and plant vigor. Field trials of these engineered plants in contaminated agricultural soils have shown complete restoration of soil functionality within three–five growing seasons, with the added benefit of producing biomass suitable for metal recovery or energy production.

For complex contamination scenarios involving mixtures of organic and inorganic pollutants, this Special Issue presents compelling evidence for integrated treatment systems that combine physical, chemical, and biological processes in optimized sequences. A comprehensive multi-year study systematically evaluated 27 different treatment combinations for soils co-contaminated with petroleum hydrocarbons and heavy metals, identifying an optimal treatment train consisting of mild chemical oxidation (using activated persulfate at controlled doses) followed by enhanced bioremediation (combining bioaugmentation with hydrocarbon-degrading consortia and metal-resistant bacteria) and concluding with assisted phytostabilization. This sequential approach achieved near-complete contaminant removal while preserving and ultimately restoring soil ecosystem functions, as evidenced by comprehensive ecological assessments measuring microbial diversity, enzyme activities, and invertebrate recolonization patterns. The research team developed a sophisticated decision-support algorithm that considers site-specific parameters (contaminant profiles, soil characteristics, climate conditions) to generate optimized treatment protocols, significantly advancing the field toward precision remediation. Another innovative solution featured in this Special Issue is the development of multifunctional reactive treatment zones that combine adsorption, chemical reduction, and biological degradation mechanisms in a single engineered system. These advanced treatment barriers incorporate hierarchical porous biochar for contaminant sequestration, bimetallic nanoparticles for reductive transformations, and bioactive zones hosting tailored microbial communities, creating a “cascade treatment” system that addresses multiple contaminant classes simultaneously. Field demonstrations across three continents have shown these systems to maintain high treatment efficiency for periods exceeding eight years, with some installations actually improving in performance over time as microbial communities adapt to the contaminant load.

The policy and regulatory dimensions of contamination management receive rigorous examination in this Special Issue, reflecting growing recognition that technical solutions alone cannot address environmental challenges without supportive governance frameworks. A comprehensive meta-analysis of international regulatory approaches to emerging contaminants revealed striking disparities in risk assessment methodologies, with particular inconsistencies in the treatment of PFAS, pharmaceutical residues, and nanomaterials. The study developed a harmonized risk assessment framework incorporating innovative elements such as high-throughput toxicogenomics, computational toxicology models, and ecosystem service valuation metrics, proposing a tiered approach that balances scientific rigor with practical implementability. Another policy-focused investigation conducted detailed socioeconomic analyses of remediation technology adoption patterns across 47 countries, identifying key barriers including upfront capital costs, technical capacity limitations, and misaligned incentive structures. The research team proposed innovative financing models such as environmental impact bonds, remediation tax credits, and land value capture mechanisms that could dramatically improve access to advanced remediation technologies in developing economies. Perhaps most significantly, several studies emphasize the critical importance of community engagement and traditional ecological knowledge in shaping effective and culturally appropriate remediation strategies, highlighting case studies where indigenous knowledge systems have contributed essential insights into contaminant behavior and ecosystem recovery patterns that were overlooked by conventional scientific approaches [10,11].

Looking toward the future, this Special Issue identifies several transformative research directions that will define the next decade of contamination science and remediation technology. The development of intelligent self-optimizing remediation systems represents perhaps the most promising frontier, combining real-time sensor networks, autonomous treatment units, and adaptive control algorithms powered by machine learning. Early prototypes of these “smart remediation” systems have demonstrated the ability to dynamically adjust treatment parameters in response to changing contaminant concentrations or environmental conditions, achieving 30–50% improvements in energy efficiency while maintaining stringent cleanup standards. Another revolutionary area involves the application of synthetic biology to create designer microbial consortia for targeted contaminant degradation, with recent breakthroughs enabling the engineering of completely novel metabolic pathways capable of breaking down previously recalcitrant compounds such as PFAS and certain chlorinated paraffins. The intersection of environmental remediation with circular economy principles is yielding particularly innovative approaches, such as electrochemical methods for simultaneous contaminant destruction and metal recovery or plasma-assisted conversion of organic pollutants into valuable chemical feedstocks. Climate change adaptation is emerging as a critical consideration in remediation design, as shifting precipitation patterns, rising temperatures, and more frequent extreme weather events alter contaminant mobility and degradation kinetics—forward-looking studies are beginning to develop “climate-resilient remediation” strategies that maintain effectiveness across a range of potential future climate scenarios.

The scientific and technological advancements presented in this Special Issue collectively represent a paradigm shift in our approach to soil and groundwater contamination, moving from compartmentalized and contaminant-specific treatments to holistic systems-based solutions that consider entire ecosystems and their services. From angstrom-scale insights into contaminant–surface interactions obtained through advanced spectroscopic techniques to landscape-scale applications of remediation technologies monitored by satellite constellations, these studies demonstrate the remarkable progress being made across all scales of environmental restoration science. However, significant challenges remain in bridging the “valley of death” between laboratory-scale innovations and full-field implementations, particularly in addressing the staggering heterogeneity of real-world environments where biological, chemical, and physical factors interact in complex and often unpredictable ways. The translation of research breakthroughs into practical solutions requires sustained truly interdisciplinary collaboration—bringing together not just environmental scientists and engineers but also data scientists, social scientists, economists, and policymakers—to develop solutions that are not only technically effective but also socially equitable and economically sustainable. The integration of indigenous knowledge systems with cutting-edge science, particularly in communities disproportionately affected by contamination, represents another crucial frontier for developing culturally appropriate and locally adapted remediation strategies.

As we progress deeper into the Anthropocene, the lessons from this Special Issue underscore the necessity of maintaining simultaneous focus on both fundamental scientific understanding and transformative technological innovation. Breakthroughs in analytical and computational techniques continue to reveal new dimensions of contaminant behavior at increasingly finer temporal and spatial scales, while advances in materials science, biotechnology, and information technology create unprecedented tools for environmental monitoring and cleanup. Perhaps most importantly, these studies collectively highlight that effective contamination management must be viewed not as an isolated technical challenge but as an integral component of sustainable development that intersects with critical issues of ecosystem health, environmental justice, climate resilience, and economic transformation. The research presented in this Special Issue of *Toxics* provides both a comprehensive snapshot of current scientific capabilities and an inspiring roadmap for future innovation, offering tangible hope that through continued scientific progress, thoughtful technology deployment, and inclusive governance approaches, we can successfully address one of the most complex environmental challenges of our era.

The extraordinary breadth and depth of these studies, spanning from molecular-level mechanistic investigations to continental-scale monitoring systems and international policy analyses, create a robust foundation for the evolving science of environmental remediation. As new classes of emerging contaminants continue to be identified and as climate change alters fundamental aspects of contaminant fate and transport, the interdisciplinary systems-based approaches showcased in this Special Issue will become increasingly essential. Future research must build upon these foundations while embracing emerging scientific paradigms, technological capabilities, and collaborative frameworks to develop resilient solutions for protecting and restoring soil and groundwater resources—the very foundation of terrestrial ecosystems and human civilization. The collective wisdom contained in these nine studies points toward a future where human activities can exist in harmony with healthy ecosystems through the judicious application of science, technology, and policy—a vision that is both imperative and achievable if we maintain this trajectory of innovation, integration, and inclusive environmental stewardship [12].

## 3. Future Development Prospects

The development of advanced detection technologies is crucial for improving the accuracy and efficiency of contaminant monitoring in soil and groundwater [13,14]. Future research should focus on integrating nanotechnology-based sensors, biosensors, and other innovative analytical techniques to enhance real-time on-site detection capabilities. The use of machine learning and data analytics can further improve the interpretation of complex environmental data, enabling more effective decision-making in contamination management. In addition, innovative remediation techniques, such as advanced oxidation processes, electrochemical remediation, and bioremediation, offer promising solutions for addressing complex and emerging contaminants. Future research should aim to optimize these technologies, reduce operational costs, and enhance their scalability and applicability in diverse environmental settings. The development of hybrid remediation strategies that combine multiple techniques can also provide synergistic benefits for treating contaminated sites. Furthermore, refining risk assessment frameworks to incorporate emerging toxicological data and better understand the long-term impacts of contaminants on human health and ecosystems is essential. The application of approaches such as the Threshold of Toxicological Concern (TTC) can aid in prioritizing remediation efforts and managing risks associated with low-level exposures. Additionally, site-specific risk management strategies that consider ecological, social, and economic factors are crucial for sustainable remediation outcomes.

It is also important to strengthen policies and regulations to address emerging contaminants and promote sustainable remediation practices. Future efforts should focus on updating existing regulations to incorporate new scientific findings and fostering international cooperation to harmonize regulatory frameworks. Public engagement and stakeholder involvement are also essential for ensuring the effective implementation of remediation strategies. Raising public awareness and promoting education on the risks associated with soil and groundwater contamination can foster community support for remediation efforts. Educational programs and communication strategies targeting diverse audiences can enhance public understanding and participation in environmental management initiatives.

## Data Availability

Not applicable.
